# Supplementary material for: The Systems Biology Research Tool: evolvable open-source software
Source: BMC Syst Biol. 2008 Jun 29;2:55. doi: 10.1186/1752-0509-2-55 (PMC2446383; doi:10.1186/1752-0509-2-55)
Supplement: Additional file 1 — SBRT Archive. An archive of the current version of the Systems Biology Research Tool. [file 1752-0509-2-55-S1.zip › sbrt-1.4.0/doc/developers_guide/api/sbrt/kernel/math/algebra/class-use/DoubleMatrix.html]

Uses of Interface sbrt.kernel.math.algebra.DoubleMatrix


|  |  |  |
| --- | --- | --- |
| **Overview** \| **Package** \| **Class** \| **Use** \| **Tree** \| **Deprecated** \| **Index** \| **Help** | |  |
| PREV   NEXT | **FRAMES**    **NO FRAMES**     **All Classes** |  |


---


## **Uses of Interface sbrt.kernel.math.algebra.DoubleMatrix**

| Packages that use DoubleMatrix | |
| --- | --- |
| **sbrt.kernel.fba.comp** | Provides classes and interfaces for performing Flux Balance Analysis computations. |
| **sbrt.kernel.fba.proc** | Provides classes and interfaces for executing Flux Balance Analysis processes. |
| **sbrt.kernel.math.algebra** | Provides classes and interfaces for creating objects relevant to algebra. |
| **sbrt.shell.mng.fba** | Provides classes and interfaces for creating Flux Balance Analysis process managers. |

Uses of DoubleMatrix in sbrt.kernel.fba.comp

| Methods in sbrt.kernel.fba.comp with parameters of type DoubleMatrix | |
| --- | --- |
| `void` | `FbaOptComputer.setMatrixElements(DoubleMatrix<?,?> elements)`             Sets the specified elements of the program matrix to those provided. |

Constructors in sbrt.kernel.fba.comp with parameters of type DoubleMatrix

`FluxCapComputer(Fluxome fluxome,
DoubleMatrix<?,java.lang.String> cycleMatrix)`
  
          Constructs a new flux cap computer from the cycles contained
in the provided fluxome.

Uses of DoubleMatrix in sbrt.kernel.fba.proc

| Method parameters in sbrt.kernel.fba.proc with type arguments of type DoubleMatrix | |
| --- | --- |
| `void` | `CycleIdProcess.run(ResultsManager<DoubleMatrix<?,java.lang.String>> resultsManager)`             Runs this process and adds the generated results to the provided results manager via its `ResultsManager.addData(Object)` method. |
| `void` | `ExtremeCurrentProcess.run(ResultsManager<DoubleMatrix<?,java.lang.String>> resultsManager)`             Runs this process and adds the generated results to the provided results manager via its `ResultsManager.addData(Object)` method. |

Uses of DoubleMatrix in sbrt.kernel.math.algebra

| Subinterfaces of DoubleMatrix in sbrt.kernel.math.algebra | |
| --- | --- |
| `interface` | `DoubleMatrixBuilder<R,C>`             This interface is used to represent mutable matrices whose elements are strictly double precision numbers. |
| `interface` | `SmdMatrix<R,C>`             This interface is used to represent sparse mutable matrices containing strictly double precision numbers as elements. |
| `interface` | `SparseDoubleMatrix<R,C>`             This interface is used to represent sparse matrices whose elements are strictly double precision numbers. |

| Classes in sbrt.kernel.math.algebra that implement DoubleMatrix | |
| --- | --- |
| `class` | `AbstractMdMatrix<R,C>`             This abstract class is a skeleton implementation of `DoubleMatrixBuilder`. |
| `class` | `AbstractSmdMatrix<R,C>`             This abstract class is a skeleton implementation of `SmdMatrix`. |
| `class` | `DoubleMatrixWrap<R,C>`             This class is used to wrap mutable matrices as an immutable `DoubleMatrix`. |
| `class` | `HashMatrix<R,C>`             This class is a `java.util.LinkedHashMap` based implementation of `SmdMatrix`. |
| `class` | `RowOrTreeMatrix<R extends Comparable<? super R>,C extends Comparable<? super C>>`             This class is a `java.util.TreeMap` based implementation of `SmdMatrix`. |
| `class` | `SparseDoubleMatrixWrap<R,C>`             This class is used to wrap mutable matrices as an immutable `SparseDoubleMatrix`. |
| `class` | `TreeMatrix<R extends Comparable<? super R>,C extends Comparable<? super C>>`             This class is a `java.util.TreeMap` based implementation of `SmdMatrix`. |

| Methods in sbrt.kernel.math.algebra that return DoubleMatrix | |
| --- | --- |
| `DoubleMatrix<R,C>` | `DoubleMatrixWrap.getColumn(java.lang.Object columnIndex)`             Returns the column corresponding to the provided column index. |
| `DoubleMatrix<R,C>` | `DoubleMatrix.getColumn(java.lang.Object columnIndex)`             Returns the column corresponding to the provided column index. |
| `DoubleMatrix<R,C>` | `DoubleMatrixWrap.getRow(java.lang.Object rowIndex)`             Returns the row corresponding to the provided row index. |
| `DoubleMatrix<R,C>` | `DoubleMatrix.getRow(java.lang.Object rowIndex)`             Returns the row corresponding to the provided row index. |

| Methods in sbrt.kernel.math.algebra with parameters of type DoubleMatrix | |
| --- | --- |
| `void` | `AbstractMdMatrix.setElements(DoubleMatrix<? extends R,? extends C> m)`             Sets the corresponding elements of this matrix to those of the provided matrix. |
| `void` | `DoubleMatrixBuilder.setElements(DoubleMatrix<? extends R,? extends C> m)`             Sets the corresponding elements of this matrix to those of the provided matrix. |

Constructors in sbrt.kernel.math.algebra with parameters of type DoubleMatrix

`DoubleMatrixWrap(DoubleMatrix<R,C> m)`
  
          Constructs a new wrapper around the provided matrix.

Uses of DoubleMatrix in sbrt.shell.mng.fba

| Methods in sbrt.shell.mng.fba with parameters of type DoubleMatrix | |
| --- | --- |
| `void` | `ExtremeCurrentManager.Rm.addData(DoubleMatrix<?,java.lang.String> results)`             Adds data to this results manager. |

---


|  |  |  |
| --- | --- | --- |
| **Overview** \| **Package** \| **Class** \| **Use** \| **Tree** \| **Deprecated** \| **Index** \| **Help** | |  |
| PREV   NEXT | **FRAMES**    **NO FRAMES**     **All Classes** |  |


---
